# High-grade hemorrhoids requiring surgical treatment are common after laparoscopic ventral mesh rectopexy

**DOI:** 10.1007/s10151-016-1432-8

**Published:** 2016-02-16

**Authors:** J. J. van Iersel, H. A. Formijne Jonkers, P. M. Verheijen, W. A. Draaisma, E. C. J. Consten, I. A. M. J. Broeders

**Affiliations:** Department of Surgery, Meander Medical Centre, Maatweg 3, 3813 TZ Amersfoort, The Netherlands; Institute of Technical Medicine, MIRA, Twente University, Enschede, The Netherlands

**Keywords:** Hemorrhoids, Hemorrhoidectomy, Laparoscopic ventral mesh rectopexy, Rectal prolapse, Recurrence

## Abstract

**Purpose:**

To describe patients developing grade III and IV hemorrhoids requiring surgery after laparoscopic ventral mesh rectopexy (LVMR) and to explore the relationship between developing such hemorrhoids and recurrence of rectal prolapse after LVMR.

**Methods:**

All consecutive patients receiving LVMR at the Meander Medical Centre, Amersfoort, the Netherlands, between 2004 and 2013 were analyzed. Kaplan–Meier estimates were calculated for recurrences.

**Results:**

A total of 420 patients underwent LVMR. Sixty-five of these patients (actuarial 5-year incidence 24.3, 95 % confidence interval (CI) 18.6–30.0) developed symptomatic grade III/IV hemorrhoids requiring stapled or excisional hemorrhoidectomy. Re-do surgery for recurrent grade III/IV hemorrhoids was required for 15 of the 65 patients (actuarial 5-year recurrence rate 40.6, 95 % CI 23.2–58.0) after the primary hemorrhoidectomy. Three of the 65 patients developed an external rectal prolapse (ERP) recurrence and eight an internal rectal prolapse (IRP) recurrence. This generated a 5-year recurrence rate of 25.3 % (95 % CI 0–53.9) for ERP recurrence and 24.4 % (95 % CI 9.1–39.7) for IRP recurrence. The rest of the LVMR cohort not receiving additional surgery for hemorrhoids (*n* = 355) showed significantly lower actuarial 5-year ERP (0.8 %, *p* = 0.011) and IRP (11 %, *p* = 0.020) recurrence rates.

**Conclusion:**

High-grade hemorrhoids requiring surgery may be common after LVMR. The development of high-grade hemorrhoids after LVMR might be considered a predictor of rectal prolapse recurrence.

## Introduction

Disorders of the pelvic floor, including urinary and fecal incontinence, pelvic organ prolapse, obstructed defecation and chronic pelvic pain, are socially disabling conditions. In the Western world, this pathology is common, affecting more than 40 % of the middle-aged and older women, with a lifetime risk of undergoing surgery of 10–20 % [1, 2]. The rectum is often involved in this multi-organ problem [3]. Various conditions including rectoceles, internal and external rectal prolapse may cause fecal incontinence and the obstructed defecation syndrome (ODS) [4].

Laparoscopic ventral mesh rectopexy (LVMR) is increasingly applied for the treatment of external rectal prolapse (ERP) and internal rectal prolapse (IRP). This technique proved to be safe and effective in terms of functional results in large cohorts of patients [5–8]. For prolapse surgery, the recurrence rate is a key indicator of success. ERP recurrence is easily assessed, but diagnosing IRP recurrence remains challenging. One hypothesis is that IRP could be the cause of high-grade hemorrhoids (III and IV), but the development of such hemorrhoids after LVMR is not well known [9]. In the past 25 years, the incidence of high-grade hemorrhoids has been quoted as up to 18 % after different types of rectopexy [10–25], but specific literature regarding LVMR is scarce. Only a handful of relatively small series mention the incidence [5, 26–28], but none of these discuss the issue in depth. The aim of this study, therefore, was to identify patients developing high-grade hemorrhoids requiring surgical treatment after LVMR and to explore the relationship between such hemorrhoids and the recurrence of rectal prolapse following LVMR.

## Materials and methods

### Study design

This observational cohort study was a retrospective analysis of a prospectively maintained database and was undertaken in a large teaching hospital in the Netherlands. All consecutive patients undergoing LVMR for rectal prolapse syndromes (Table 1) between March 2004 and May 2013 were analyzed.

**Table 1:** Patient characteristics, medical history and initial indications for LVMR

Patient characteristics	LVMR *N* = 420 (%)	Hemorrhoidectomy group *N* = 65 (%)
Woman/men [mean age]	404/16 [61.8]	61/4 [60.4]
*History*		
Mean para (range) [episiotomy]	2.4 (0–10) [37]^a^	2.6 (0–5) [12]^b^
Hysterectomy	139 (33.1)	47 (72.3)
Cystopexy	39 (9.3)	4 (6.2)
Anterior colporrhaphy	56 (13.3)	13 (20.0)
Sphincter operation	6 (1.4)	0
Other abdominal surgery	137 (32.6)	23 (35.4)
Rubber band ligation before LVMR [second session]	28 (6.7) [7]^c^	3 (3.1) [2]^d^
Pre-hemorrhoidectomy—*before initial LVMR*	20 (4.8)	2 (3.1)
RBL between LVMR and hemorrhoidectomy [second RBL]	39 (9.3) [13]	4 (6.2) [1]
*Indication for initial LVMR*		
ERP	55 (13.1)	5 (7.7)
IRP^e^ and/or symptomatic rectocele	266 (63.3)	44 (67.7)
IRP^e^ and/or symptomatic rectocele with enterocele	99 (23.6)	16 (24.6)

*LVMR* laparoscopic ventral mesh rectopexy, *ERP* external rectal prolapse, *IRP* internal rectal prolapse, *RBL* rubber band ligation

^a^In 25 patients

^b^In 7 patients

^c^Two patients underwent a third and a fourth session

^d^One patient underwent a third and a fourth session

^e^Oxford rectal prolapse grade III/IV

### Patients and evaluation

Postoperatively, all patients were prescribed a laxative (Macrogol 3350/electrolytes, Movicolon^®^, Norgine Limited, Mid Glamorgan, UK). Follow-up after LVMR was carried out according to a standardized protocol and performed at 6 weeks after surgery by one of the three participating experienced pelvic floor surgeons (P.V., E.C. and I.B.). At the 6-week follow-up, the presence of hemorrhoids, recurrence of rectal prolapse, incontinence and constipation was assessed. All patients were asked to return in the event of anorectal complaints. Patients were examined for hemorrhoids in the standing and lithotomy position using leg supports, both in rest and during straining. In addition, proctoscopy was performed. Hemorrhoids were graded using the Goligher classification [29]. Patients with grade II and III hemorrhoids were treated with rubber band ligation (RBL) first. Persisting symptomatic grade III/IV hemorrhoids (‘high grade’) were considered an indication for surgery, but results of LVMR were awaited for at least 10 weeks. Lower grades of hemorrhoids were not operated on. ERP recurrence was clinically assessed. IRP recurrence was defined as Oxford rectal prolapse grade III/IV IRP with symptoms of obstructed defecation or fecal incontinence. Most of these patients had a coexisting rectocele or enterocele. A dynamic MRI of the pelvic floor was done on all patients suspected of an IRP recurrence. A large part of the study cohort (most patients operated from 2004 to 2011) had participated in a previous study about the outcomes of LVMR, and therefore, a longer follow-up period was available for these patients [7]. For those patients not included in this previous study, no additional effort was made to systematically follow them up. Kaplan–Meier curves were used to establish whether there was a difference in outcome between the two groups.

### Surgical technique

All laparoscopic ventral mesh rectopexies were performed according to the technique described by D’Hoore et al. [6]. All meshes used were synthetic. Either a stapled hemorrhoidectomy (SH) or a traditional excisional hemorrhoidectomy (TEH) was performed. Where a SH was not technically possible, a TEH was done. Surgery was performed by, or under direct supervision of, one of the three pelvic floor surgeons (P.V., E.C. and I.B.). Operations were performed under general or spinal anesthesia. The patients were placed in the lithotomy position using adaptive leg supports with swing stirrups. The PPH 03 stapler produced by Ethicon (EndoSurgery, Cincinnati, Ohio, USA) was used for SH. The stapled procedure had been previously standardized and was performed according to the technique described by Singer et al. [30]. Excisional hemorrhoidectomy was performed according to standard protocol [31].

### Statistical analysis

Statistical Package for the Social Science Advanced version 20.0 (IBM Corp., Armonk, NY, USA) was used for statistical analysis. Data are presented as percentage, median and range. Because of differences in follow-up between patients, the Kaplan–Meier method was used to estimate the incidence of postoperative high-grade hemorrhoids and recurrence rates at various points in time. The risk estimates after a period of 5 years are shown in the text. *p* < 0.05 was considered statistically significant.

## Results

### Patients characteristics

Before LVMR, twenty-eight patients (6.7 %) underwent RBL for grade II hemorrhoids and 20 patients (4.8 %) underwent a hemorrhoidectomy for grade III/IV hemorrhoids.

A total of 420 patients (16 men; 404 women) underwent LVMR. Indications for surgery were ERP (*n* = 55, 13.1 %), IRP (Oxford rectal prolapse grade III/IV) and/or symptomatic rectocele (*n* = 266, 63.3 %) and IRP and/or symptomatic rectocele combined with enterocele (*n* = 99, 23.6 %). General patient characteristics are presented in Table 1.

### Follow-up

The median follow-up after LVMR was 16.0 months (range 0.4–93.7). Three hundred and ninety-one patients (93.1 %) were available for follow-up after the standardized outpatient visit at 6 weeks postoperatively. Nine patients (2.1 %) died of causes unrelated to the LVMR within the study period.

**Fig. 1:**
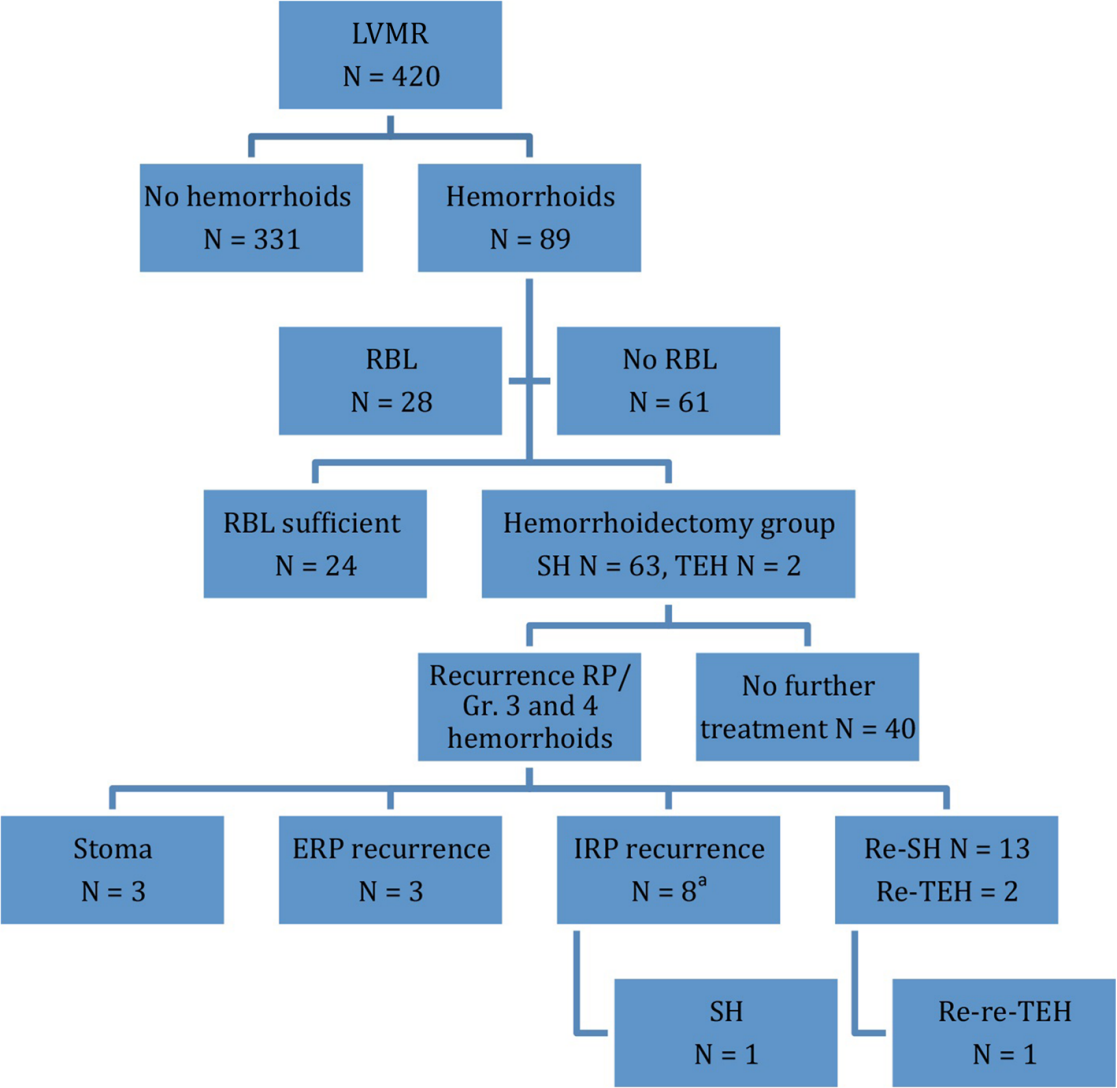
Flowchart. *LVMR* laparoscopic ventral mesh rectopexy, *RBL* rubber band ligation, *SH* stapled hemorrhoidectomy, *TEH* traditional excisional hemorrhoidectomy, *RP* rectal prolapse, *Gr.* grade. *ERP* external rectal prolapse, *IRP* internal rectal prolapse. ^a^Four of these patients received an re-SH first

During follow-up after LVMR, 89 patients required treatment for hemorrhoids, of which 24 were treated sufficiently by RBL. The remaining 65 patients (Kaplan–Meier estimate of 24.3 % at 5 years, 95 % CI 18.6–30.0, Table 2) received surgical treatment for symptomatic grade III/IV hemorrhoids and are referred to as the ‘hemorrhoidectomy group’ (63 SH, 2 TEH). Four of the hemorrhoidectomy group (6.2 %) received RBL between LVMR and the hemorrhoidectomy without sufficient result (flowchart Fig. 1). The median duration between LVMR and hemorrhoid surgery was 6.2 months (2.5–45.3).

**Table 2:** Kaplan–Meier estimates (%) for incidence and recurrence of gr. III/IV hemorrhoids and recurrence of rectal prolapse in the hemorrhoidectomy group (*n* = 65) and the non-hemorrhoidectomy group (*n* = 355) at various time points

Kaplan–Meier estimates % [CI]	Years		
	1	3	5
Gr. III/IV hemorrhoids after LVMR	16.5 [CI 12.4–20.6]	22.2 [CI 17.1–27.3]	24.3 [CI 18.6–30.0]
*Recurrence high*-*grade hemorrhoids*			
Hemorrhoidectomy group (*n* = 65)	31.2 [CI 16.9–45.5]	35.2 [CI 19.7–50.7]	40.6 [CI 23.2–58.0]
*External rectal prolapse recurrence*			
Hemorrhoidectomy group (*n* = 65)	0	2.0 [CI 0–5.9]	25.3 [CI 0–53.9]
Non-hemorrhoidectomy group (*n* = 355)^a^	0.8 [CI 0–2.0]	0.8 [CI 0–2.0]	0.8^b^ [CI 0–2.0]
*Internal rectal prolapse recurrence*			
Hemorrhoidectomy group (*n* = 65)	1.9 [CI 0–5.6]	20.2 [CI 6.5–33.9]	24.4 [CI 9.1–39.7]
Non-hemorrhoidectomy group (*n* = 355)^a^	2.1 [CI 0.3–3.7]	5.7 [CI 2.0–9.4]	11.0 [CI 4.3–17.7]

*CI* 95 % confidence interval, *LVMR* laparoscopic ventral mesh rectopexy, *gr.* grade

^a^This cohort contains the 420 patients receiving a LVMR minus the patients developing postoperative high-grade hemorrhoids; 420–65 = 355

^b^One ERP recurrence after 64.6 months

### High-grade hemorrhoids recurrence

Fifteen patients of the hemorrhoidectomy group (15/65) needed re-do surgery (*n* = 13 SH) for recurrent grade III/IV hemorrhoids after a median of 8.3 months (1.5–40.5) after the primary hemorrhoidectomy. The estimated percentages (Kaplan–Meier) were 31.2 % after 1, 35.2 % after 3 and 40.6 % after 5 years (95 % CI 23.2–58.0, Table 2). One patient received an excisional hemorrhoidectomy after twice a SH in a period of 8.9 months. This was the only patient receiving more than two hemorrhoidectomies after LVMR.

### Rectal prolapse recurrence—ERP

**Fig. 2:**
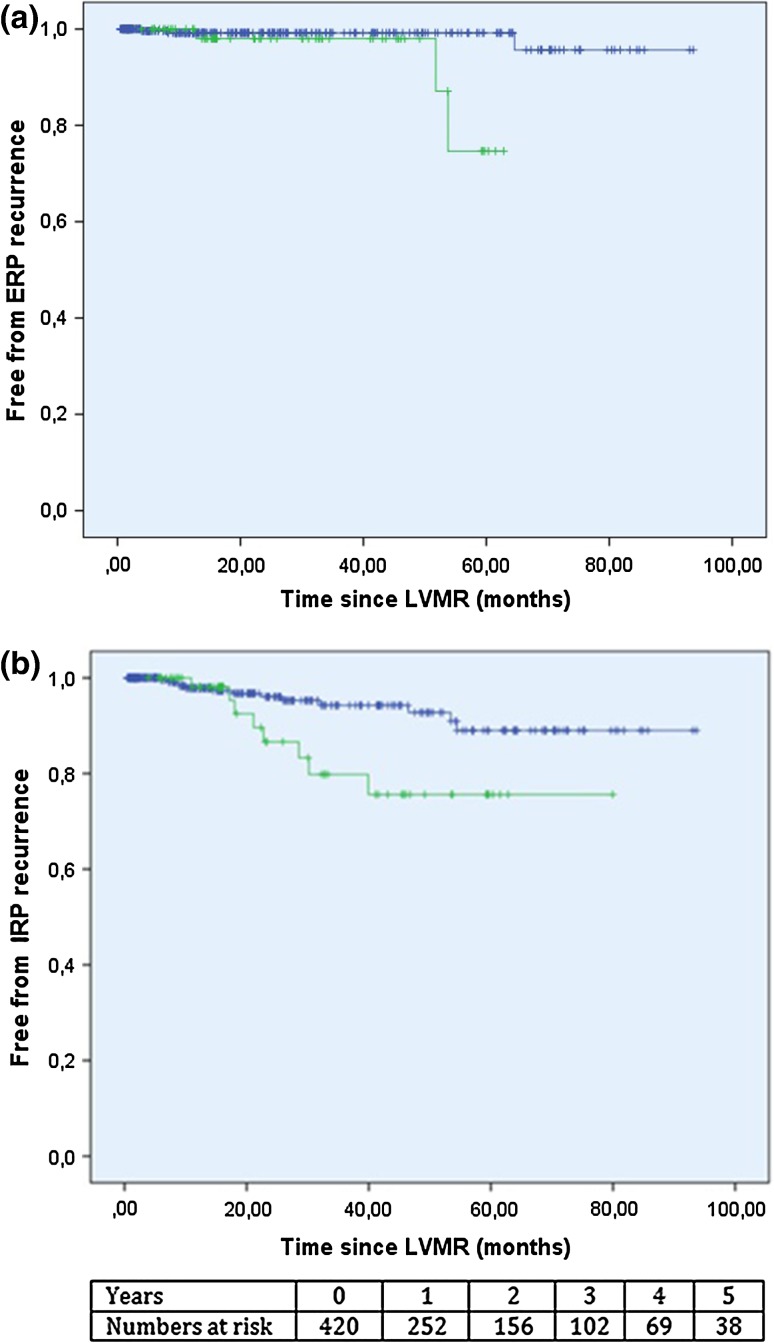
**a** Kaplan–Meier curve for ERP recurrence (cohort *n* = 420). The *green line* represents the cohort developing high-grade hemorrhoids after LVMR (‘hemorrhoidectomy group,’ *n* = 65), and the *blue line* represents the rest of the LVMR cohort not developing high-grade hemorrhoids after LVMR (‘non-hemorrhoidectomy group’ *n* = 355, *p* = 0.011). **b** Kaplan–Meier curve for IRP recurrence (cohort *n* = 420). The *green line* represents the cohort developing high-grade hemorrhoids after LVMR (‘hemorrhoidectomy group,’ *n* = 65), and the *blue line* represents the rest of the LVMR cohort not developing high-grade hemorrhoids after LVMR (‘non-hemorrhoidectomy group,’ *n* = 355, *p* = 0.020). The duration of event-free survival was measured from date of LVMR to the time of the event (complete) or the last follow-up (censored) for both curves. At the bottom of the figure, a table with the number of patients left for analysis per year is presented

In the hemorrhoidectomy group, three patients (3/65) developed a clinical full-thickness external prolapse generating a recurrence percentage (Kaplan–Meier estimates) 0 % after 1, 2.0 % after 3 and 25.3 % after 5 years (95 % CI 0–53.9). Two of these patients underwent re-do LVMR and the third patient declined surgery. The ERP recurrence rate (Kaplan–Meier estimates) in the group of patients who did not received additional surgery for hemorrhoids after LVMR (‘non-hemorrhoidectomy group,’ *n* = 355) was 0.8 % after 5 years (95 % CI 0–2.0). This is significantly (*p* = 0.011) lower compared to the hemorrhoidectomy group (*n* = 65, Fig. 2a and Table 2).

### Rectal prolapse recurrence—IRP

Additionally, eight patients of the hemorrhoidectomy group (8/65) were diagnosed with an IRP recurrence. The Kaplan–Meier estimates for IRP recurrence were 1.9, 20.2 and 24.4 % after 1, 3 and 5 years (95 % CI 9.1–39.7). One patient decided against surgery and the rest received re-do rectopexy. One patient required a SH 9 months after the re-do rectopexy. Due to persistent fecal incontinence without curative surgical options, a stoma was created in a further three patients.

The estimated IRP recurrence percentage after 5 years was significantly lower (11 %, *p* = 0.020, 95 % CI 4.3–17.7) in the non-hemorrhoidectomy group (*n* = 355) compared to the hemorrhoidectomy group (*n* = 65, Fig. 2b and Table 2).

## Discussion

The exact incidence of high-grade hemorrhoids following LVMR is not known. Our study found a high actuarial 5-year incidence of 24.3 %. In the literature, only four articles report on this issue, quoting a lower incidence varying from 1.6 to 5 % (Table 3) [5, 26–28]. However, these studies describe the outcome of LVMR rather than focus on the development of postoperative high-grade hemorrhoids. Of the two studies with substantial follow-up, the study of D’Hoore et al. only followed up the patients by telephone and Slawik et al. did not perform an anorectal examination after 3 years [5, 27]. In the other two studies, the follow-up period was substantial shorter [26, 28]. As a result, in these studies the occurrence of high-grade hemorrhoids after LVMR may have been underestimated. Furthermore, in the reported studies the main indication for LVMR was ERP, whereas in our study 86.9 % of the patients presented with IRP. Also, two studies combined the LVMR with other procedures (e.g., STARR or resection rectopexy) [27, 28]. This heterogeneity among studies might explain the differences in reported percentages of high-grade hemorrhoids after LVMR. In our study, 53.8 % of the hemorrhoidectomy group (35/65) suffered from ODS complaints before LVMR and most of them had a long history of straining and incomplete evacuation. After LVMR, still 15 patients of the hemorrhoidectomy group (15/65, 23.1 %. *p* = 0.004) reported persisting ODS complaints. Other studies quote a slightly lower figure (up to 19 %) of patients suffering from persisting ODS after LVMR [5–8]. The high incidence of grade III/IV hemorrhoids after LVMR and the high recurrence rate of grade III/IV hemorrhoids after hemorrhoidectomy might be the result of persistent straining. The actuarial 5-year recurrence rate of 40.6 % for grade III/IV hemorrhoids after hemorrhoidectomy was very high compared to several randomized controlled trials showing recurrence rates from 0 to 5 % for both excisional as stapled hemorrhoidectomy [32–35]. It is also possible that some of the patients in the hemorrhoidectomy group were not properly assessed before LVMR. Possibly, some patients underwent LVMR for symptomatic IRP combined with a rectocele, whereas retrospectively the symptoms might have been caused mostly by a mucosal prolapse. Consequently, it could be that high-grade hemorrhoids following LVMR might be attributed to residual mucosal prolapse in some cases. In these patients, it seems that LVMR repairs the rectal prolapse, but fails to correct the mucosal prolapse. If so, a different operation instead of LVMR (e.g., STARR) might have been more appropriate. Because of the retrospective character of this study, it is unfortunately not possible to verify if mucosal prolapses were missed before LVMR.

**Table 3:** Available literature concerning high-grade hemorrhoids requiring surgery after LVMR

First author	No. of patients	Indication LVMR	Follow-up in months (median)	High-grade hemorrhoids after LVMR	Treatment
D’Hoore [5]	42	ERP	61	1 (2.4 %)	SH
Slawik^a^ [27]	80	44 ERP 36 IRP	54	4 (5 %)	3 SH, 1 TEH
Wijffels [26]	80	ERP	23	2 (2.5 %)	1 SH, 1 STARR
Randall^b^ [28]	190	ERP	29	3 (1.6 %)	3 SH

*LVMR* laparoscopic ventral mesh rectopexy, *SH* stapled hemorrhoidectomy, *STARR* stapled transanal rectal resection

^a^Seven patients underwent a laparoscopic resection rectopexy, and 74 females underwent concurrent posterior colporrhaphy and vaginal sacrocolpopexy

^b^LVMR was combined with Orr–Loygue (*n* = 3), anterior colporrhaphy (*n* = 7), posterior STARR (*n* = 10) and SH (*n* = 2)

The role of mucosal prolapse in hemorrhoidal disease is in debate. Gaj et al. [36] showed that 40 % of the proctologists do not consider mucosal prolapse as independent from hemorrhoidal disease in a national survey. We believe that mucosal prolapse is an integral part of the hemorrhoidal disease. However, whether mucosal prolapse is a completely different entity or not, with excising a circumferential band of excessive rectal mucosa and submucosa and interrupting the blood supply of the superior hemorrhoidal artery proximal to the hemorrhoidal tissue, the clinical condition is treated either way.

In addition, it is worth noting that the hemorrhoidectomy group includes more patients with a history of hysterectomy (72.3 % vs. 33.1), re-do of the initial LVMR (12.3 % vs. 8.8 %) and number of past episiotomies (8.8 % vs. 18.5 %) than the non-hemorrhoidectomy group. All these variables might constitute an increased risk of developing high-grade hemorrhoids after LVMR. No other differences worth mentioning were found between the groups.

The incidence of recurrence of rectal prolapse in the hemorrhoidectomy group was also high, with an actuarial 5-year ERP recurrence rate of 25.3 % and an actuarial 5-year IRP recurrence rate of 24.4 %. In contrast, the non-hemorrhoidectomy group (*n* = 355) showed significantly lower actuarial 5-year ERP (0.8 %, *p* = 0.011) and IRP (11 %, *p* = 0.020) recurrence rates (Fig. 2a/b; Table 2). The literature quotes similar incidences to our non-hemorrhoidectomy group with rates varying from 1.6 to 4.8 % [5, 6, 28, 37] for ERP and from 0 to 15 % for IRP [8, 38]. This could suggest that patients with high-grade hemorrhoids after rectopexy are susceptible to developing a rectal prolapse recurrence after LVMR. The hemorrhoidectomy group seems to contain a cohort of patients with persisting symptoms possibly not well responding to the standard therapy. Both high recurrence rates of rectal prolapse and grade III/IV hemorrhoids are indicative. The three patients requiring a stoma due to persistent fecal incontinence support this impression. It may be that high-grade hemorrhoids after LVMR are a sign of laxity of (a part of) the posterior compartment and represent the first stage of a continuum, eventually developing into rectal prolapse. Consequently, the findings of this study could suggest that the development of high-grade hemorrhoids following LVMR might be considered predictive of a rectal prolapse recurrence. In order to exclude a rectal prolapse recurrence, additional radiological imaging should be considered when a patient presents with grade III/IV hemorrhoids following LVMR. Unfortunately, our data did not offer a clear explanation for the relationship between post-LVMR high-grade hemorrhoids and rectal prolapse recurrence. As there is no literature available on this potential relationship, it would be an interesting topic for future studies.

LVMR has been performed in our hospital since 2004. Analysis shows that the occurrence of post-LVMR hemorrhoidectomy is fairly stable over the years. This indicates that there is probably no learning curve problem, or sign of insufficient repair. This is supported by the rates for ERP and IRP recurrence in the non-hemorrhoidectomy group which are comparable to the contemporary literature.

A limitation of this paper is the differences in follow-up between patients. Although the Kaplan–Meier method yields appropriate estimates for recurrence rates at various points in time, underestimation remains possible. When we compared patients receiving extended follow-up in the context of a previous study (*n* = 149) [7] with those followed up according to the standardized postoperative protocol (*n* = 271), the risk of high-grade hemorrhoids was somewhat higher with the standardized postoperative follow-up. However, estimates were unstable and the difference was not statistically significant (*p* = 0.149). In the standardized follow-up protocol, the probability of the patient presenting at the outpatient clinic after the standard 6 week postoperative control might be related to the degree of postoperative complaints, and therefore, selection bias may have occurred.

## Conclusion

High-grade hemorrhoids requiring surgery may be common after LVMR. The development of high-grade hemorrhoids after LVMR might be considered a predictor of rectal prolapse recurrence.
